# The role of institutional factors and cognitive absorption on students’ satisfaction and performance in online learning during COVID 19

**DOI:** 10.1371/journal.pone.0269609

**Published:** 2022-06-22

**Authors:** Sameera Butt, Asif Mahmood, Saima Saleem

**Affiliations:** 1 Institute of Quality & Technology Management, University of the Punjab, Lahore, Pakistan; 2 Department of Innovation and Technology Management, College of Graduate Studies, Arabian Gulf University, Manama, Bahrain; Fondazione Ugo Bordoni, ITALY

## Abstract

With the rise of the Covid-19 pandemic, there has been a severe negative impact on all aspects of life, whether it be a job, business, health, education, etc. As a result, institutions, schools, colleges and universities are being shut down globally to control the spread of Covid-19. Due to this reason, the mode of education has a dramatic shift from on-campus to online learning with virtual teaching using digital technologies. This sudden shift has elevated the stress level among the students because they were not mentally prepared for it, and hence their academic performance has been adversely affected. So, there needs to figure out the underlying process to make online learning more productive. Thus, to obtain this objective, the present study has integrated the modified Technology Acceptance Model (TAM), Task Technology Fit Model (TTF), DeLone and McLean Model of Information Systems Success (DMISM) and Unified Theory of Acceptance and Use of Technology (UTAUT) model. A sample of 404 students was obtained, where 202 students were from the top ten public sector universities, and 202 were from the top ten private sector universities of Punjab. Structural Equation Modelling (SEM) was used to analyze the hypothesized framework using AMOS. The results reveal that institutional factors positively impact students’ performance mediated by user satisfaction and task technology fit. Similarly, institutional factors affect performance through mediation by user satisfaction and actual usage in sequence. Cognitive absorption was used as a moderator between institutional factors and user satisfaction. In the end, theoretical and practical inferences have also been discussed.

## 1. Introduction

The COVID‐19 pandemic constrained universities all around the globe to shut down their campuses inconclusively and move their instructive activities onto online modes. The deadly virus discovered in China in December 2019 outspread throughout the world in no time, and hence was pronounced as a pandemic by the World Health Organization on March 11, 2020. In the spring of 2020, most universities were supposed to close their campuses and move their entire academic programs online [1]. Universities were unprepared for such a change from traditional classroom instruction to entirely online education delivery [2]. But enormous technological evolution in the past few decades has proved immensely useful during this pandemic [3, 4]. There were online portals to promote online education [5]. Numerous tools used by the institutions to deliver online education during this pandemic are Zoom, Google Meet, Google Classroom, Microsoft Teams, Google Forms, docs and sheets, and so on. Despite this, educators and students encountered various administrative, economic, technological and social issues [6, 7].

The deadly pandemic and the ensuing lockdowns had a significantly destructive effect on people’s mental health all over the world [8, 9]. Such mental and psychological issues regularly impede many students from adjusting to online instruction. Besides, all students also do not have the same kind of access to and understanding of emerging technology. While these differences were present before, the COVID 19 pandemic has brought this digital gap to light [10]. Because of the raging pandemic, [1] was the first to explain how universities relocated from classroom-based education to online education. Although many countries had extensive online education infrastructure before the pandemic [11], still no university was prepared for a complete transition to online learning. Observational examinations have discovered that students prefer learning in actual study halls to online sessions [12]. Students miss time with their friends in classrooms, labs and admittance to the library [13].

In any case, students feel that online classes assisted them with continuing their studies during the pandemic [11]. Universities are now using novel techniques and strategies to ensure that their students continue to receive a quality education [14]. According to [15], the digital divide existed before the pandemic, but it escalated. Online education needs consistent access to digital technology. Students with limited access to new technology and those unfamiliar with them have difficulty adjusting to online education. Moreover, some students live in distant areas and have a problem attending online classes from their homes [13]. Furthermore, because of the unanticipated transformation from traditional face-to-face on-campus courses to online learning, students, teachers and educational institutions face many challenges. Among these, the most critical challenge is implementing high-quality online education systems, adapting the latest online learning technologies, and providing high-quality education. So, because of these numerous difficulties, there was a negative effect on students’ academic performance and subsequently, their grades declined [16]. Therefore, a question arises, ‘what needs to be done to enhance the satisfaction level and academic performance of the students with the help of online education systems’?

Before analyzing the essential components in online learning that can elevate the satisfaction level and academic performance of the students, it is required to initially understand the fundamental theory of e-learning and the various constituents of e-learning. Online education is a system that imparts education with the help of the internet while utilizing laptops, smartphones, desktops, tablets, etc. [17]. The governments of various countries worldwide are dispensing their maximum efforts to encourage the usage of the latest technology in online education systems [18]. It is also believed to have many advantages, like it saves time, supports interpersonal communion, enhances learning efficiency, delivers advanced education and learning, provides authentic information, saves cost, supports adjustable location choice, and minimizes time-based problems associated with on-campus learning [19–21]. Thus, it is evident from these advantages that e-learning is effective and advantageous for the health of the learners, instructors, and relevant staff’ in the time of the pandemic covid-19.

Thereby, many researchers have made significant contributions in creating different hypothetical ideas and developing various models in the context of information systems to ascertain and explain the attitude and behavior of the users with the relevant technology. The essential models found in the literature relevant to information systems are the Theory of Reasoned Action (TRA) [22], Theory of Planned Behavior (TPB) [23], Technology Acceptance Model (TAM) [24], Task Technology Fit (TTF) model [25], DeLone and McLean Model of Information Systems Success (DMISM) [26, 27] and Unified Theory of Acceptance and Use of Technology (UTAUT) model [28]. But the discernment of utilization of information systems has been greatly neglected in these models and their corresponding hypothetical conceptions [29], with the DMISM as an exception that determines the usage of information technology by examining the influence of overall quality on user satisfaction, actual system usage and performance. So, it is immensely utilized to evaluate the proficiency and productiveness of information systems [30].

Thus, in a majority of the research-oriented online learning, variables were obtained from these models to assess the effectiveness of online learning and its numerous established frameworks. Moreover, multiple factors affect the satisfaction level and academic performance in online learning. Among these factors, institutional factors also play a significant role in enhancing the performance level of the students. According to [31], the institutional environment and infrastructures significantly affect students’ performance. According to [32], reducing the class size could improve learning, while sufficient research equipment and teaching content could significantly enhance the students’ performance. [33] asserted that an improved physical environment offers comfort, security, and a better knowledge of courses and is impactful in the form of higher learning and performance. Another factor recognized as significant in online learning is cognitive absorption, an individual characteristic identified by several experts as necessary in using and influencing technology. In recent Malaysian research on digital libraries [34] it was observed that there is a significant positive association between cognitive absorption and user satisfaction. Likewise, in Spain, [35] observed that cognitive absorption had a substantial positive link with perceived usefulness, perceived ease of use, and user satisfaction. [36] examined the influence of online learning platforms on user satisfaction through cognitive and emotional engagement. Similarly [37], in his research work, determined the influence emotions have on online learning. [38] evaluated the factors that affect online learning utilization, like effort expectancy, social influence, facilitating conditions, performance expectancy etc.

Similarly, [39] determined the effect of compatibility and task technology-fit as mediators on the usage of e-learning with the help of the Information System Success Model (ISSM). Hence, the researchers have analyzed the association of online learning with numerous variables. Yet, there are still certain gaps present in the literature that are required to be determined and investigated, such as there are only limited studies based on online learning that have analyzed the impact of institutional factors on performance impact [40–42]. To date, limited studies have been done on how institutions worldwide coped with the COVID-19 outbreak and prepared for it. A considerable number of assessments and forecasts have been published on the likely influence of COVID-19 on higher education. However, there have been few quantitative published studies on this subject, and the research aimed at examining how online distance learning implemented during the COVID-19 lockdown has influenced the teaching approach in higher education is still being developed. Moreover, minimal research has analyzed student performance in online distance learning during lockdown to prior learning in face-to-face classes. Likewise, contradictory studies have been observed based on user satisfaction, actual system usage and performance impact. Research studies that advocate the existence of this relationship are [43–45], yet studies like [46, 47] analyzed that there is no such association that is present among user satisfaction and performance impact. This contradiction additionally presents an advantage in observing the significance or insignificance of the effect of user satisfaction on actual system usage and performance. Besides, the presence and contribution of mediators and moderators in the context of online learning models have been slightly addressed, like the contribution of human-relevant attributes such as cognitive absorption is scarcely being described.

Hence, specific research questions are constituted by examining the issues encountered by the online education system in the time of pandemic Covid-19 and the gaps observed in the prior literature work. They are (a) By what means task technology fit (i.e., academic tasks of the students are fit with the respective e-learning system) may influence institutional factors and user satisfaction to enhance the performance of the learners during COVID-19? Likewise, (b) By what means actual usage (i.e., frequency of using the online education system by the learners) may influence institutional factors and user satisfaction to enhance the performance of the learners during COVID-19? (c) Can cognitive absorption of learners elevate the satisfaction level of learners pertaining to the quality of the online education system?

Thus, a research framework has been established in this study derived from the consolidation of the (TTF), (DMISM) models with the inclusion of essential components, institutional factors derived from the facilitating conditions variable of the (UTAUT) model and cognitive absorption derived from perceived ease of use and perceived usefulness variables in (TAM) model. In the TTF model, the significant construct is the task technology fit and its relationship with performance impact, but it neglects its association with the constructs of overall quality, user satisfaction, and actual system usage. On the other hand, the DMISM model specifies the relationship between the construct’s overall quality, user satisfaction, actual usage, and performance impact constructs but neglects the task technology construct present in the TTF model. The UTAUT model aims to describe user intentions for the usage of information systems and associated usage behavior. Finally, the technology acceptance model (TAM) describes how users tend to accept and utilize a particular technology. So depending on the above-discussed literature, six variables were selected to constitute an online learning framework where institutional factors served as an independent variable in the framework, user satisfaction, actual usage, task technology fit, performance impact acted as dependent variables, while cognitive absorption used as a moderator between institutional factors and user satisfaction.

Furthermore, the association between institutional factors and performance impact is being analyzed in two ways: via serial mediation of user satisfaction and actual usage; and via serial mediation of user satisfaction and task technology fit in series and cognitive absorption acts as a moderator on the association between institutional factors and user satisfaction. This research contributes to the literature by providing empirical evidence on the causal influence of online education on student academic performance during the COVID-19 crisis using data from top universities in Punjab, Pakistan. Moreover, this research has also analyzed the online learning satisfaction level and performance among the students after the COVID-19 breakout caused an abrupt transition from face-to-face to online learning. Furthermore, we contribute to the empirical evidence on the influence of instructional models on student academic performance in online learning during Covid-19. This research also examined various factors at the institutional level that have a considerable impact on the satisfactional level and academic performance of the students in the online learning system. Moreover, this research study is important as it will present guidance to the experts more extensively on how the academic performance of the students’ can enhance by the usage of online learning during the time of the Covid-19 pandemic.

## 2. Literature review and hypotheses development

### 2.1. Literature review

There has been an enormous increase in the average number of students acquiring distance education in the past few years, especially online education for their higher education. According to research by [48], the most significant necessity to successfully implement distance education is to achieve student satisfaction. Therefore, keeping in view the importance of the satisfaction level of students in online education, it has become the primary concern of the institutions to determine the factors that elevate the level of student satisfaction. Among many factors, institutional factors are considered quite significant for enhancing the satisfaction level of the students. Institutional factors consist of assistance plans or specifications delegated as standards, conventions, or principles for student engagement to fulfill the requirements set up for graduation [49]. As the institutions devote their earnest efforts to achieving the success of their online education programs, the institutions should prioritize and give serious consideration to stabilizing and enhancing their support services, the weakening of which may become an obstacle for them in attaining their desired goals. This study will consider four important institutional factors: institutional support, administrative support, instructional support, and technical support. As [50] recommended, an effective distance education system needs a considerable extent of institutional support directed towards enhancing distance learning and education quality. Technical support is regarded as one of the significant components, and this support is provided by highly skilled and trained people who are hardware and software experts [51]. It is the technical assistance provided to the students while using the distance learning technology. Another critical institutional factor is administrative support which refers to services such as online student registration, record maintenance, online class scheduling, providing course relevant information, training etc. [52]. Instructional support is referred to as the guidance by the instructor for learning that comprises answers to student queries, rectifying the misconceptions of the students, delivering clear content to the students and providing productive feedback to the learners on their class performance and assignments.

In the context of online learning, user satisfaction is described as the degree to which online learning students realize satisfaction in their sole decision to depend on such services and how effectively they fulfill their requirements [35, 53, 54]. With reference to this study, TTF is defined as the extent to which an online learning system corresponds to users’ interests, fits their tasks, and fulfills their requirements. It is also referred to as the extent to which technology facilitates the users to accomplish their coursework or online learning activities. Actual usage is the extent of frequent usage of technology and the number of times it has been used [55]. Performance impact is defined as the degree to which using a system leads to the enhancement of work quality by assisting users to accomplish their tasks rapidly, enhancing job conduct, eradicating errors and improving job efficacy [56, 57]. In this research, the performance impact is referred to as the extent to which an online learning system influences the academic performance of the students in relation to asset reservation, proficiency, productiveness and knowledge attainment [58]. Cognitive absorption is defined as the extent to which users consider the system useful and are eager to use it again [44]. It is also referred to as a state in which the user is deeply involved with the software or a holistic experience of the student with IT, i.e., the comprehensive experience of an individual with the information technology as he/she uses the internet as well as online learning as related to attention, time and enjoyment [59]. Cognitive absorption denotes one of the kinds of intrinsic motivation, in which ‘‘the behavior is enacted for itself, to come across the inherent contentment and satisfaction”.

In research by [60], it was observed that student satisfaction is affected by a positive attitude towards technology and an independent learning approach. In their research, [61] analyzed the association between the perceived level of presence, perceived usefulness and ease of use with student satisfaction and persistence by obtaining data from an online university in South Korea. Their research highlighted that cognitive presence, teaching presence, and perceived usefulness and ease of use are regarded as the essential components for acquiring student satisfaction. [62] recommended that all universities have a flexible institutional structure to incorporate online learning technology to enhance learning effects. So apart from other factors that affect student satisfaction level and performance, institutional factors are also significant, and it comprises areas that impact student retention, and these can or cannot be changed by the institution [63]. Therefore, institutional factors have a significant impact on elevating the satisfaction level with regard to distance education, as has been discussed in detail, and many institutional factors emphasized that it might affect the student’s understanding as well. The instructors and instructional formulators must arrange for a favorable learning environment [64]. The support provided to the students proves to be one of the essential elements that impact students’ success in the context of online learning [65, 66]. Research work by [67] analyzed the recognition factors of online education systems, and they observed that cognitive absorption has a positive impact on perceived ease of use and perceived usefulness. Moreover, in relevance to Information Technology, cognitive absorption was observed to have an important influence on user satisfaction in the occurrence of regular website usage [68] in the context of usage of online learning systems [35, 69, 70] and usage of social networking websites like Facebook [71]. [34] analyzed that cognitive absorption positively influences user satisfaction.

Higher education institutions and governments are trying to introduce online learning around the world, as analyzed by [19]. In addition to this, [72] depicted their model as significant in making e-learning more beneficial and determining its success. The results demonstrated that the strength of e-learning positively influenced the performance and satisfaction level of the individual. [73] observed that technology characteristics and task characteristics of substantial open online courses positively anticipated task technology fit. Apart from this, perceived relatedness, perceived competence and social recognition remarkably determine the behavioral intention of students. Furthermore, this research also analyzed that cognitive absorption acts as a moderator on user satisfaction, where cognitive absorption was found to have a significant positive impact on student satisfaction [19].

### 2.2. Hypotheses development

#### 2.2.1. Institutional factors and user satisfaction

According to previous studies, institutional factors substantially influence learner satisfaction. A research study was carried out in the UK to determine the impact of institutional factors on learner satisfaction in online learning. It was observed that the quality of teaching and learning is an essential component in postgraduate student retention and satisfaction [74]. Research by [75] proposed that with the increase in the intensity of institutional support services in online learning, the magnitude or extent of student satisfaction also increases. Other studies also endorse that institutional factors impact user satisfaction [76–78]. Therefore, it can be inferred that institutional factors significantly intensify student satisfaction in online learning.

Various studies have recognized technical support as a significant component that leads to student satisfaction. Research findings by [79] depict that students’ willingness to accept or refuse an information system is significantly affected by the quality of technical support. If the users face any issue with the online learning system and practically do not receive any assistance from the support, then the students perceive that it is simply time waste to use the particular online learning system, and hence they may stop using it. So the more efficient will be the technical support, the more the students will be satisfied with the system. Research by [80] emphasizes that support of the online system administration is believed to have a significant role in user satisfaction with technology usage. The course instructor essentially provides instructional support, however, technology can be utilized to deliver support to the students individually and improve their instructional environment [81]. Research by [82] indicates that instructional support significantly impacts student satisfaction.

An essential factor in IS practices and a significant success element used in adopting a new system is the primary user satisfaction [30, 83]. According to the present research, it is being suggested that the higher the level of support by the institutional factors of the online learning system, the higher will be the satisfaction level of the students. The more these technologies will help the students accomplish their online assigned tasks, the more they will evolve into prerequisites for accomplishing their educational commitments. There it can be hypothesized that

H_0_: Institutional factors do not have a significant influence on user satisfaction.
H_1_: Institutional factors have a significant influence on user satisfaction.

#### 2.2.2 User satisfaction and task technology fit

Various studies have affirmed the significant role of user satisfaction in IS practices in different conditions and technological applications. Task Technology Fit is perceived as a highly imperative component when examining technology applications in various organizations [84]. Many studies have analyzed the association between task technology fit and user satisfaction, and they examined that a substantial direct interrelationship is present among these variables [42, 57, 84–89]. Hence, in this study, it is presumed that user satisfaction has a significant positive influence on task technology fit, given the presumption that the higher the quality level of the technological system used in online learning, the higher will be the satisfaction level of the students, and more they will acknowledge the technology as essentially fit to fulfill their online pedagogical activities.

H_0_: User satisfaction does not positively predict task technology fit
H_2_: User satisfaction positively predicts task technology fit

#### 2.2.3 Task technology fit and performance impact

In the past few years, vigorous progression has been observed in technology and the inclusion of several novel systems; particular emphasis is directed towards the realization of the usage of the technological system in relevance to elevation in the performance level of users to assess system productivity [30, 90, 91]. Numerous research work exists in the literature that has inductively assessed the relationship between task technology fit and performance impact and observed that task technology fit has a significant positive influence on performance impact [42, 43, 57, 84–89]. It has been further analyzed that performance of students’ in relevance to proficiency and productiveness is enhanced due to task technology fit [92].

Therefore, the following hypothesis is deduced:

H_0_: Task technology fit does not positively predict performance impact.
H_3_: Task technology fit positively predicts performance impact.

#### 2.2.4 User satisfaction and actual usage

One more important component in technology-intended studies is the knowledge of technology utilization by the users. Many research works have been conducted to investigate the relationship between user satisfaction and actual system usage, with the conclusion that the user satisfaction construct has a considerable impact on actual system usage [93–96]. In fact, the time span of technology utilization by users is enhanced due to user satisfaction [90]. The important element in this supposition is also user satisfaction, as it is an obvious fact that when the satisfaction level of the user is high, the actual system usage will also be enhanced. Hence, it can be hypothesized that:

H_0_: User satisfaction does not positively predict actual usage of the system
H_4_: User satisfaction positively predicts actual usage of the system

#### 2.2.5 Actual usage and performance impact

The relationship between actual usage of the system and performance impact is one more critical aspect in the frame of reference of technology utilization [97]. Few studies have tried hard to minimize the gap by doing considerable work on the relationship between actual usage of the system and performance impact [57, 98]. A quantitative study by [95] analyzed that the actual system usage has an important effect on performance. Moreover, research work centered on information systems has emphasized that actual usage of a system has a positive influence on performance construct [54, 84, 98–102]. Hence, this relationship illustrates that the students will use the more persistently the online learning system to complete their academic tasks, the more it will contribute to their improved academic performance.

So, it is hypothesized that:

H_0_: Actual usage of the system does not positively predict the performance impact
H_5_: Actual usage of the system positively predicts performance impact

#### 2.2.6 Mediating role of user satisfaction

User satisfaction is favorably influenced by institutional factors [75, 77, 78], and user satisfaction has a considerable positive influence on task technology fit [39]. Hence, it is being advocated that institutional factors positively impact task technology fit through user satisfaction. Besides, it is determined from the prior discussed literature that the time span of technological system utilization is enhanced due to user satisfaction [58], and user satisfaction is correspondingly affected by the institutional factors [19, 102].

Based on these arguments, the following hypotheses are proposed:

H_0_: Institutional factors do not positively predict task technology fit through the mediating role of user satisfaction
H_2a_: Institutional factors positively predict task technology fit through the mediating role of user satisfaction
H_0_: Institutional factors do not positively predict actual usage of the system through the mediating role of user satisfaction
H_4a_: Institutional factors positively predict actual usage of the system through the mediating role of user satisfaction

#### 2.2.7 Mediating role of task technology fit

As it was observed that with the increase in the potency of the institutional support services, the intensity of student satisfaction is also enhanced, leading to task technology fit in the sequence. According to [90], user satisfaction is an essential factor that significantly impacts users’ performance. User satisfaction and task technology fit are the vital components of this presumption. Nevertheless, as [43] observed, an important association was determined between task technology fit and performance impact. This relationship was practically verified, and it was deduced that task technology fit positively influences performance impact [43]. Indeed, the academic performance of the students in relevance to their efficacy and productivity is enhanced due to task technology fit [103], and the technology fit fulfills the needs of the students when they are highly satisfied, as analyzed by [39], consequently because of a higher level of institutional factors.

Hence, it is deduced that:

H_0_: Institutional factors do not positively predict performance impact through the mediating role of user satisfaction and task technology fit in the sequence.
H_3a_: Institutional factors positively predict performance impact through the mediating role of user satisfaction and task technology fit in the sequence.

#### 2.2.8 Mediating role of actual usage

It was analyzed that an increase in the level of institutional factors of the system directs towards the elevated satisfaction level of the students and has an indirect impact on actual usage of the system through user satisfaction. User satisfaction and actual usage are the vital elements in this supposition [58]. Moreover, past research also suggests that actual usage of the system has a significant effect on performance [84], and actual system usage is itself affected by user satisfaction [58]. User satisfaction is essentially influenced by institutional factors [104]. Therefore, institutional factors certainly affect performance impact through user satisfaction and actual system usage in sequence. Therefore,

H_0_: Institutional factors do not positively influence performance impact through the mediating role of user satisfaction and actual usage of the system
H_5a_: Institutional factors positively influence performance impact through the mediating role of user satisfaction and actual usage of the system

#### 2.2.9 The moderating role of cognitive absorption

Cognitive absorption comprises three discrete measures: inherent interest, curiosity and observation center [105]. Cognitive absorption has been found to positively influence the online learning system’s perceived usefulness [106] and perceived ease of use [67, 107]. Cognitive absorption is a kind of intrinsic motivation [108]. Users submerge in the usage of a particular IT system for their personal objectives and feel a sense of joy and pleasure when they use it. If an online education system satisfies the intrinsic motivation and imparts a feeling of enjoyment to the students, they will keep using it and spend more time on it.

Hence, the satisfaction throughout the usage of the online learning system arises from cognitive absorption, which enables the students to fully concentrate and immerse in their educational activities. A research study by [109] highlights that cognitive absorption is positively associated with user satisfaction. In this research, cognitive absorption focuses on how the students feel completely involved with online learning while using it with full joy and are fully attentive. Numerous studies emphasize that cognitive absorption is a significant factor in user satisfaction [19, 69, 71, 110]. Moreover, research studies by [34, 35, 111] determined that cognitive absorption has an essential impact on user satisfaction. Therefore, it can be inferred that cognitive absorption significantly influences user satisfaction in online learning, i.e., the greater the level of cognitive absorption of the students using the online learning system, the higher their satisfaction level will be. Thus, it is hypothesized that:

H_0_: Cognitive absorption does not moderate the relationship between Institutional factors and User Satisfaction.
H_6_: Cognitive absorption moderates the relationship between Institutional factors and User Satisfaction.

**Fig 1:**
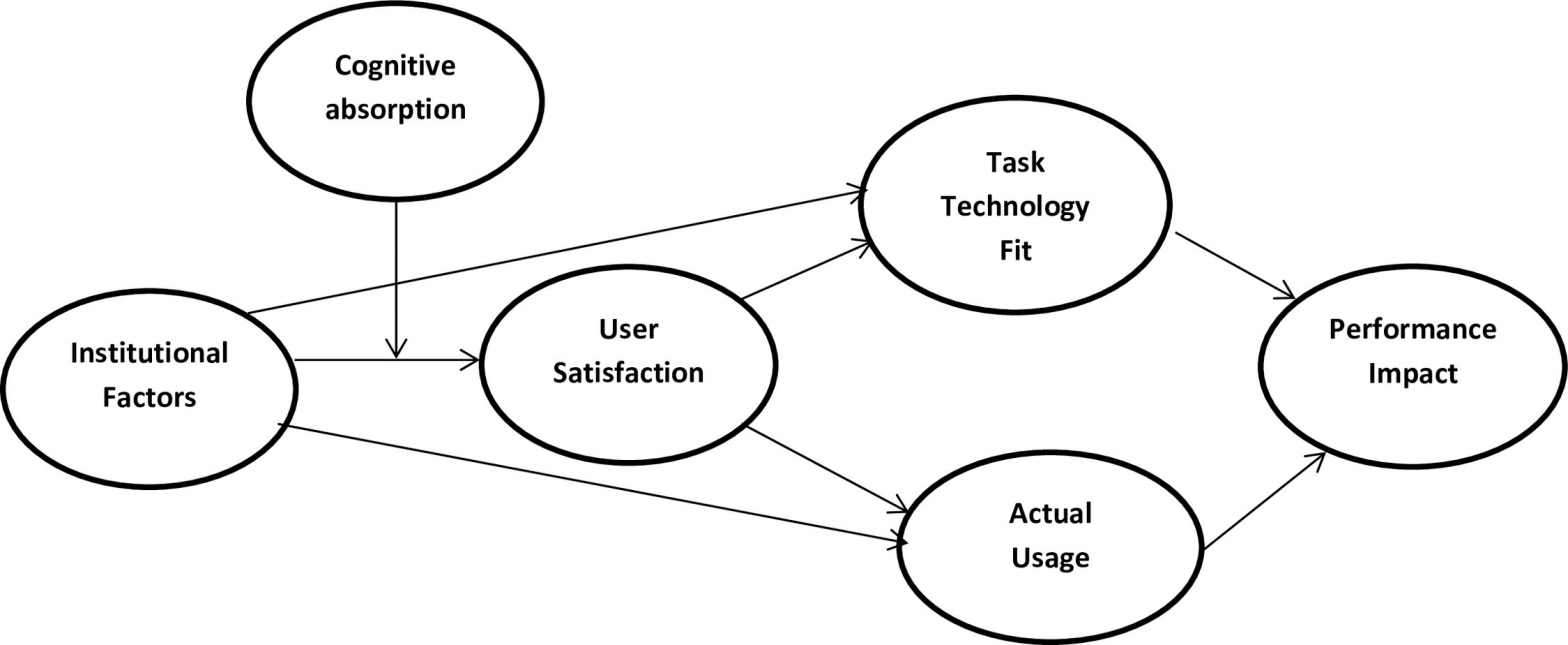
The theoretical framework for the current study.

Based on these hypotheses, Fig 1 depicts the relationships among the variables of the study.

## 3. Methodology

### 3.1 Ethics statement

The Institution’s Ethical Evaluation Committee (NML-ERC/2021-02) provided ethical review and approval for this study on human subjects. Furthermore, the respondents gave their written informed consent to take part in the research.

### 3.2 Research design

Structural Equation Modelling (SEM), a multivariate statistical analysis technique, has been used to analyze the structural associations and empirically test the formulated hypotheses with the help of Analysis of Moment Structures (AMOS^®^) 24 software. SEM comprises two components: Confirmatory Factor Analysis (CFA) is utilized to assess the measurement model among the observed and latent variables. The second is Path Analysis (PA), which is used to fit the structural model and the latent variables. CFA is used for testing the validity of indicators, whereas the path analysis technique signifies the mode where a particular latent variable directly or indirectly causes a change in the other latent variable. This 2-step approach demonstrates that the structural model makes use of only those constructs that possess a suitable measure. A goodness-of-fit index was estimated in SEM between the conceptual model and the sample data. Three different measures were used to assess the goodness of fit of the measurement model and the structural model: relative Chi-square ratio over the degree of freedom (χ^2^/DF), Goodness of Fit Index (GFI) and Root Mean Square Error Approximation (RMSEA).

### 3.3 Sample and procedure

The population for this research study is students of the top ten public sector universities and top ten private universities in Punjab as ranked by HEC Pakistan. The age group of the students ranged from 20 to 40 years. They were students of bachelor, master, Ph.D. degrees or any other diploma courses in the universities. Quota sampling was used to obtain data from the students. The data were gathered by distributing a self-administered questionnaire in hard form and forwarding the questionnaires to students at various universities via email and posting the questionnaire link on the enlisted universities’ Facebook pages. Google form questionnaire was used as a tool for online data collection. The response was only requested from the students studying through some online mode. This was ensured by asking them the screening question. The total number of questionnaires distributed among the students was 1000. Of the 1000 questionnaires, 416 responses were received, while 404 were selected for further analysis, excluding 12 due to incomplete or improper information. Therefore, the valuable response percentage of the data collection was 40.40%.

The first section of the survey was about demographics. Table 1 shows that 60 percent of respondents were male students. Moreover, 12.10% of the students were under 21 years, 79% were between the 21–30 age group, and 8.10% were between the 31–40 age group. Furthermore, the universities’ percentile responses ranged from 5.0 to 12.90%. Intermediate students made up 0.7 percent of the respondents, while bachelor students made up 53.20%, master’s students made up 32.90%, 8.70% were Mphil students, Ph.D. students made up 2.70%, and other students made up 1.50% of the overall responses.

**Table 1:** The demographics of the respondents.

		Frequency	Percent	Cumulative Percent
**Gender**				
	2) Females	163	40.35	100
	Total	404	100.00	
**Age**				
	1) 20 or less	49	12.10	12.10
	2) 21–30	319	79.00	91.10
	3) 31–40	36	8.90	100
	Total	404	100	
**University**				
	BZU-Bahauddin Zakariya University	30	7.40	
	COMSATS Institute of Information Technology	30	7.40	
	FC-Forman Christian College & University, Lahore	31	7.70	
	GCU Government College University	52	12.90	
	GIFT	30	7.40	
	IIUI-International Islamic University Islamabad	1	.20	
	IUB-The Islamia University of Bahawalpur	30	7.40	
	LUMS-Lahore University of Management Sciences	20	5.00	
	NUST	30	7.40	
	PMAS PMAS ARID UNIVERSITY	30	7.40	
	PU-University of the Punjab	30	7.40	
	RIPHAH	30	7.40	
	UOL-University of Lahore	30	7.40	
	UOS-University of Sargodha	30	7.40	
**Education**	Total			
	1) Intermediate	3	.70	.70
	2) Bachelors	215	53.20	54.10
	3) Masters	133	32.90	87.10
	4) MPhil	35	8.70	95.80
	5) Ph.D.	11	2.70	98.50
	6) Others	7	1.73	100
	Total	404	100	

Furthermore, as [112] suggested, common method variance (CMV) was provisionally carried out. CMV might exist when all the scale items are estimated with one questionnaire, and the whole data are gathered at one time. CMV also occurs when the relationship between two constructs is distorted; to put it another way, CMV constructs a methodical covariance despite the real relationship among the scale items. The perplexity of the scale items, the respondents’ incapability, double-barreled research items, incapability of the respondents to examine the research topic, less participation of the respondent in the topic, arrangement of the scale items, the respondent’s tendency to provide intense responses, and so on are all sources of CMV. Consequently, incorrect estimates of convergent validity and reliability in the research can be caused by the changed values of the observed correlations and related indicators.

CMV can be handled in two ways: one by using procedural amendments and the other by using different statistical techniques. The most efficient way to reduce CMV using procedural remedies is to identify the resemblance between predictor and criterion variable measurements and then limit or eliminate them. To avoid CMV, the researchers apply procedural remedies in the early stages of questionnaire development. A common procedural remedy is using more than one information source to get data for the model’s constituents. Another approach would be to use clear, concise, and suitable language to avoid misinterpretation of scale items by participants and reduce the possibility of undesired results. Researchers that use procedural remedies may indeed be able to minimize, if not eliminate, the possible effects of CMV in their research results. In these scenarios, they may be more willing to choose one of the statistical techniques available. Furthermore, the researcher has access to a wide variety of statistical methods for preventing CMV. The most widely used of these methods is Harman’s single-factor test, also known as Harman’s one-factor test [113]. In this procedure, the researcher enters the scale items into a single exploratory factor analysis and then examines the unrotated factor solution to find the count for the constituents with eigen values larger than one, representing cumulative variation. It is presumed here that if CMV exists, then just one component is responsible for more than half of the covariance amongst scale items. All 37 items in this study were subjected to a single exploratory factor analysis using this approach, and an unrotated factor analysis was discovered, accounting for only 28.79 percent of the total. As a result, the CMV output confirmed that the sample data did not contain any CMV bias.

### 3.4 Measurement scale

Measurement scales previously established were utilized in the current study for data collection, as presented in S1 Appendix. Each scale item was assessed on a seven-point Likert scale (1 Strongly Disagree and 7 Strongly Agree). The scale for Institutional factors was adopted from [76, 114]. It has 13 items and a reported value of Cronbach alpha of 0.926. A sample item of the scale is “I knew where to ask for help when I had any technical issues”. User Satisfaction has 3 items taken from [19]. The alpha value for this scale is 0.915. A sample item is “My decision to use online learning was a wise one”. Task Technology was adopted from [90]. It has 3 items with a reported alpha value of 0.911, and a sample item is “Online learning fits with the way I like to learn and study.” Actual usage was acquired from [39], and has 2 items and the alpha value for this scale is 0.818. A sample item is “on average, how much time do you spend per week using the online learning?”. Performance Impact was adopted from [19]. This scale has an alpha value of 0.959 with 10 items, and a sample item is “online learning helps me accomplish my tasks more quickly”. Cognitive absorption was taken from [59]. This scale has 6 items, and a sample item is “While on the Web, I am immersed in the task I am performing.”, while it has an alpha value of 0.893. The overall summary of all the items has been reported in Table 2.

**Table 2:** Measurement scales and corresponding references for all the constructs.

Construct	Measurement Scale	References
Institutional Factors	13 items	[76, 114]
User Satisfaction	3 items	[104]
Cognitive Absorption	6 items	[115]
Task Technology Fit	3 items	[90]
Actual Usage	2 items	[39]
Performance Impact	10 items	[104]

## 4. Data analysis and results

A highly efficient software Analysis of a Moment Structure (AMOS) was employed in the present study for performing data analysis. It is statistical software that uses innovative mechanisms for conducting structural equation modeling (SEM). It has a graphical interface that can be operated easily, produces an explicit research model for the researchers, creates quality illustrations for presentation in the publication, and calculates the most authentic numeric values.

### 4.1. Descriptive analysis

The values measured for descriptive analysis for Institutional factors, User Satisfaction, Cognitive absorption, Task Technology Fit, Actual Usage, and Performance Impact are shown in Table 3. Institutional factors had a mean of 4.8, and a standard deviation of 1.4; User Satisfaction had a mean of 4.7 and a standard deviation of 1.4. The mean of Cognitive Absorption was found to be 4.7 with a standard deviation of 1.2. Moreover, the mean for Task Technology Fit was 3.9, and the standard deviation turned out to be 1.8. For Actual Usage, the mean value was 4.7, and the value for standard deviation was 1.5. Lastly, the mean value for Performance Impact was 4.8, and the value obtained for standard deviation was 1.6. It indicates that the coefficient of variation (CV = Mean/Std Dev) is not very large, and there is not much dispersion in the data, which suggests that the responses obtained are reliable.

**Table 3:** Descriptive statistics.

	N	Minimum	Maximum	Mean	Std.Deviation	Skewness		Kurtosis	
						Statistic	Std.Error	Statistic	Std.Error
Cognitive absorption	404	1.25	6.75	4.72	1.29	.002	.121	-.700	.242
Institutional factors	404	1.00	7.00	4.85	1.41	-.594	.121	-.167	.242
User Satisfaction	404	1.33	7.00	4.75	1.41	-.001	.121	-.713	.242
Performance Impact	404	1.00	6.89	4.77	1.57	-.632	.121	-.663	.242
Task Technology Fit	404	1.00	6.67	3.88	1.78	.021	.121	-1.083	.242
Actual usage	404	1.00	7.00	4.67	1.47	-.739	.121	-.171	.242

As reported in Table 3, the skewness values unveil that the data has a normal distribution because the values of skewness range from − 3 and + 3, and kurtosis between − 10 to + 10, when applying structural equation modeling (SEM) [116].

### 4.2. Measurement model

**Fig 2:**
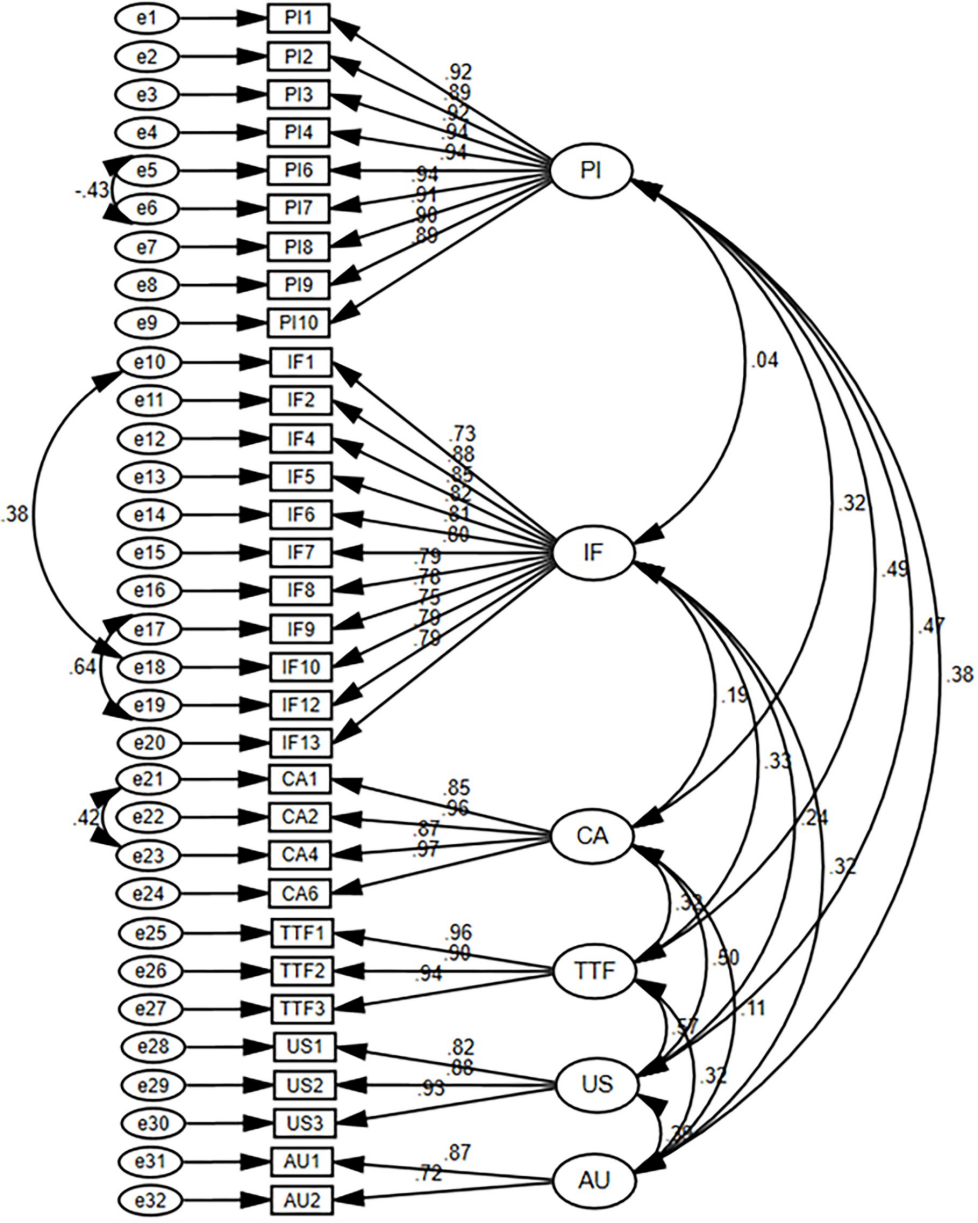
CFA for measurement model.

The measurement model represents comprehensive or implied models that relate latent variables with corresponding indicators. Moreover, it is also identified as path analysis. It helps us determine the goodness of measures of the conceptions we are intrigued by in our hypothetical model. This, in turn, allows us to assess how efficiently we can evaluate the theories to correct the errors and estimate the overall fit of these models. Confirmatory Factor Analysis (CFA) is an important method widely used to confirm a theoretical measurement model. It illustrates an interrelation between the unobserved or latent variables and observed variables or indicators. Moreover, CFA is a statistical approach that has a foundation on concepts that explain errors in the measurement and estimates the unidimensional model, and so it is suggested to carry out data analysis. The measurement model was assessed by using construct validity and reliability. Therefore, CFA was carried out by analyzing the factor constitution of the variables to evaluate the validity of the six measures, as displayed in Fig 2.

Structural Equation Modeling (SEM) used in this study is a highly efficient data analysis technique, and there is no other technique that can give us more precise results of the parameters, presuming multivariate normal data. SEM attempts to justify the applicability of a given hypothesis by assessing the impact of mediators on the relationship between an independent variable and a dependent variable. SEM was also used to examine the role of controls and moderators. Three attributes distinguish all SEM Models: evaluation of many interconnected dependency connections and the potential to describe unknown concepts in these associations, rectify measurement inaccuracies in the estimating process and create a model to describe the complete set of relationships. The major assumptions in applying structural equation modeling are: normality of the acquired data; no systematic missing data; sufficiently large sample size and correct model specification. These assumptions were checked before the application of SEM. For example, normality was assessed through Skewness and Kurtosis (see Table 3). Similarly, a sample size of 404 employed in this study is considered to be greater than the minimum threshold of 200 cases typically used in SEM studies, as recommended by [117]. Finally, we employed the Ramsey Regression Specification Error Test’s (RESET) null hypothesis of correct specification [118]. The p-value of the F stat was found to be 11.45%>5%, indicating that the functional form of the model is correct, and it does not succumb to omitted variables. Furthermore, in this study, the maximum likelihood (ML) estimation method was used, which proclaims that if all the items load significantly on their corresponding factors, then the uni-dimensionality is present for the constructs, and hence validity is exhibited. Fig 2 represents the measurement model and, composite reliability estimates for the scale reliability and results of CFA are given in Table 4.

**Table 4:** Discriminant validity.

	CR	AVE	MSV	MaxR(H)	PI	IF	CA	TTF	US	AU
**PI**	0.979	0.839	0.236	0.980	**0.916**					
**IF**	0.951	0.639	0.111	0.954	0.037	**0.799**				
**CA**	0.952	0.832	0.254	0.972	0.325***	0.192***	**0.912**			
**TTF**	0.950	0.865	0.323	0.957	0.486***	0.333***	0.319***	**0.930**		
**US**	0.910	0.772	0.323	0.924	0.467***	0.239***	0.504***	0.568***	**0.879**	
**AU**	0.776	0.636	0.148	0.808	0.376***	0.320***	0.114***	0.324***	0.385***	**0.797**

Note: For all the constructs, square roots of AVE (Average Variance Extracted) are shown as diagonal elements and inter-construct correlations are shown as off-diagonal.

#### 4.2.1 Model fit

Fit in research signifies the capability of a model to describe the data. Specifically, in CFA, a model fit demonstrates the proximity of the observed data and supplements the interrelation suggested in the theoretical model. A model with a good fit is conveniently well suited to the data, i.e., to evaluate if the model considerably fits in conformance with the data or not. Consequently, the goodness-of-fit of the model in connection to data was estimated by the application of numerous tests. So predicating on the goodness-of-fit indices, there is a proclamation of the model affirmation.

Chi-square (χ^2^) statistic denotes a test that estimates the relevance of the hypothesized model to observed data. It is used to assess the overall model fit and the variance between the sample and fitted covariance matrices. But since χ^2^ is based on the sample size, it is not employed to estimate the model fitness. However, for assessing the model fit, we calculate the χ^2^/DF, where the ratio is ≤ 3, it denotes an acceptable fit [119] and if the value is ≤ 5, it represents a reasonable fit [120]. The model used for observation in the present study has a normed chi-square (χ^2^/DF) = (936.179 /445) = 2.105 (<3.00), indicating a satisfactory fit. The goodness of fit index (GFI) is a metric for comparing the fit among the proposed model and the measured covariance matrix. According to [121], the Goodness-of-Fit Index (GFI ≤ 1) enumerates the fragment of variance constituted by the determined covariance of the population. If the value obtained is equal to 1, it will be regarded as a perfect fit. But, when the sample size increases, the GFI value is also expected to increase. When the GFI value is > 0.95, it is estimated as a good fit, and if the value of GFI is < 0.65, it is interpreted as a sustainable fit. The value of GFI obtained is 0.872, which represents an acceptable model fit. In covariance structure modeling, the root mean square error of approximation (RMSEA) and standardized root mean square residual (SRMR) is regarded as the most significant constituent. RMSEA is an indicator that measures the difference between the observed covariance matrix per degree of freedom and the predicted covariance matrix that represents the model. Whereas, the SRMR is an empirical measure of fit that is specified as the standardized difference between the actual and estimated correlations. If the value of RMSEA is <0.05, it is considered a good fit, and if the value lies between 0.08 to 0.10, it is regarded as an average fit, and the value greater than 0.10 denotes a poor fit, whereas SRMR<0.09 represents a good model fit [122]. The values obtained for RMSEA = 0.052 and for standardized RMR = .0319 illustrate reasonable unidimensionality of the constructs.

#### 4.2.2 Reliability of the variables

Reliability in research is described as the ability which the research methods to yield reliable and uniform results. In this research, Cronbach’s alpha has been used to determine the internal consistency reliability or to observe the degree to which a set of items is related. The value of Cronbach’s alpha falls between 0 and 1, and an inflated value denotes an inflated internal consistency. The Cronbach’s alpha values for the specified measures tabulated in S2 Appendix are higher than 0.70, representing the threshold value that describes convenient reliability for the measures used in this study [123].

#### 4.2.3 Construct validity

Validity is referred to as “the integrity or adequacy of a test or device in quantifying what it is devised to measure.” [124]. In the current research, construct validity was calculated after verifying discriminant validity, face validity, and convergent validity. As items for measurement were obtained from the former research, it justifies the face validity. Convergent validity was observed and estimated using the average variance extracted (AVE) and indicator reliability, which is the extent to which a measure is associated positively correlated with different measures of the same construct.

For estimating the reliability of the indicators, we employed factor loading. Construct with high loadings indicate that the associated indicators appear to have much in common exhibited by the construct [125]. Factor loadings with a value greater than 0.50 were considered highly significant [125]. It was examined that all the items seemed important with (p<0.001), and the loadings were higher than 0.5 (as illustrated in S2 Appendix), which depicts that items have satisfied all the conditions. Furthermore, all AVE values were greater than the recommended value of 0.50 [125]. Convergent validity was obtained explicitly for all constructs, and satisfactory convergent validity is exhibited in Table 4.

Similarly, discriminant validity refers to how well items distinguish across constructs or evaluate discrete ideas for the measurement model. The Fornell-Larcker criteria and the Heterotrait-Monotrait ratio (HTMT) were used for its verification.

The discriminant validity is described as the degree to which items discriminate amid constructs or assess individual conceptions for the measurement model, and it was authenticated by using Fornell-Larcker and the heterotrait-monotrait ratio (HTMT). As per the Fornell-Larcker method, the square roots of the AVEs (indicated in the diagonal of Table 4) are higher than the association between the constructs (relative row and column values). Hence, it denotes that the constructs are strongly linked with their relative indicators in contrast to other constructs in the model [124, 126], which specifies the existence of satisfactory discriminant validity [127]. The interrelationship between exogenous constructs is estimated to be lower than 0.85 [128]. Hence, we have achieved the discriminant validity for each construct in the model.

The Fornell-Larcker test was also criticized to some extent. [103] described that it fails to elaborate exactly the nonexistence of discriminant validity in general research scenarios. Hence, a substitute method was advised depending on the multitrait-multimethod matrix: HTMT—the heterotrait-monotrait ratio of correlations. Discriminant validity in the present research is assessed with the help of HTMT. The discriminant validity is likely to have some problems if the value obtained for HTMT is higher than the value of 0.90, i.e., HTMT 0.90, or the value of 0.85, i.e., HTMT 0.85. Since all the values obtained, as shown in Table 5, were smaller than the proposed value of 0.85, this signifies the attainment of discriminant validity.

**Table 5:** HTMT analysis.

	PI	IF	CA	TTF	US	AU
**PI**						
**IF**	0.039					
**CA**	0.351	0.199				
**TTF**	0.496	0.331	0.325			
**US**	0.461	0.250	0.518	0.581		
**AU**	0.383	0.320	0.138	0.326	0.393	

### 4.3. Structural model assessment

SEM analysis is the second mandatory approach in the structural equation model. It can be demonstrated after validating the measurement model by illustrating the relationship between the constructs. So, the structure model explains the relationship among the variables, possessing the particular associations among exogenous variables and corresponding endogenous variables and displaying the connection between constructs. The structural model results help us assess how closely the theory is supported by empirical data and helps to assess whether the theory is confirmed empirically [129]. The structural model’s goodness-of-fit corresponded to the CFA measurement model’s goodness-of-fit. Therefore χ^2^/df = 3.14, CFI = 0.938, and RMSEA = 0.073 in the proposed structural model. These fit indices demonstrated a good match between the predicted model and the observed data.

### 4.4. Path analysis and hypothesis testing

**Fig 3:**
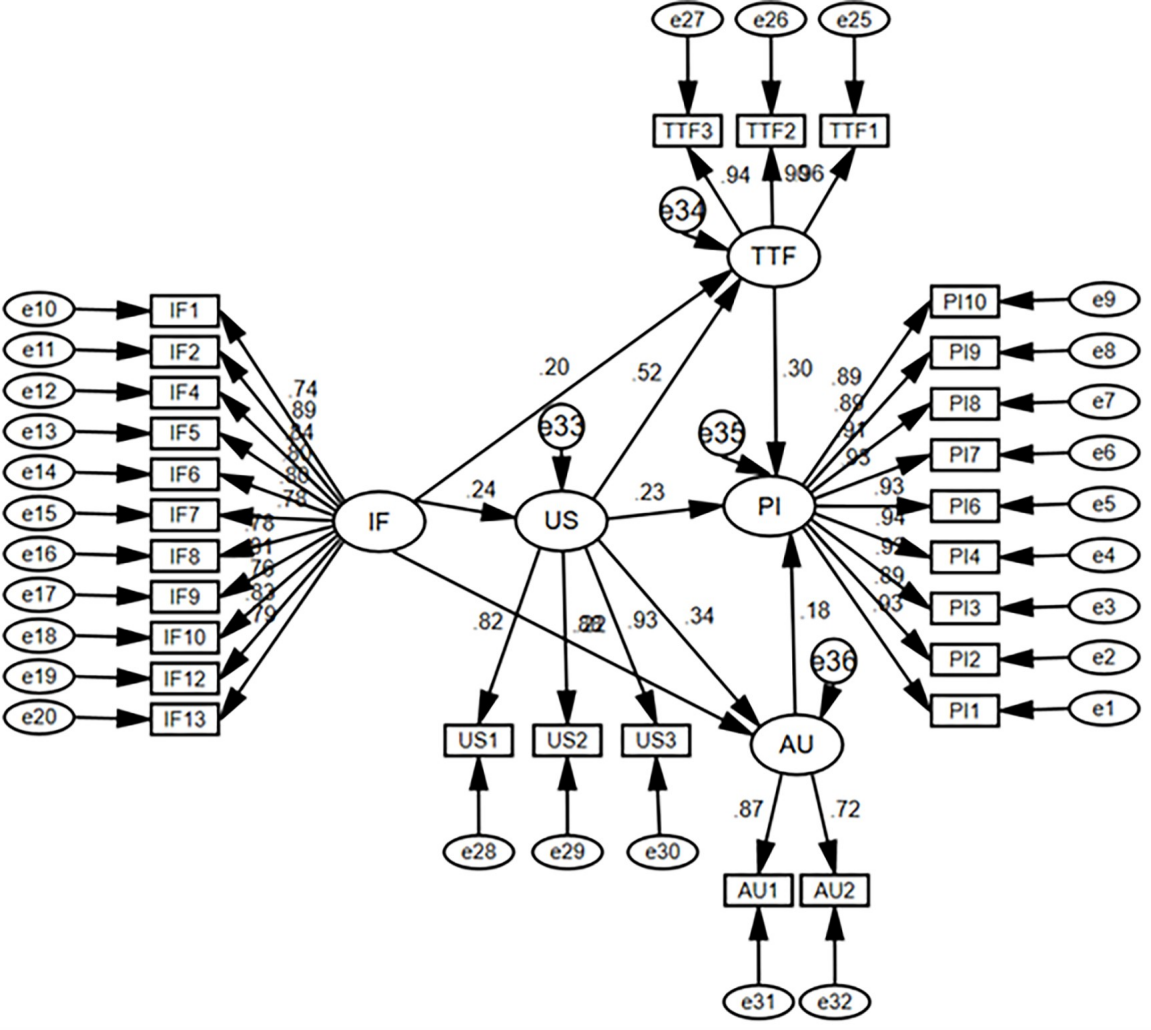
The structural model.

Exogenous variables were studied using path analysis to identify their direct and indirect effects. A path diagram in Fig 3 demonstrates the hypothesized relationship between the constructs established on results from previous literature. IF is an exogenous variable, while US, AU, TTF and PI are endogenous variables.

Bootstrapping technique tests the indirect effects in the structural models by calculating the beta (β) values, R^2^, and the respective t-values. Moreover, the p-value is used to determine the presence of the effect [130].

The structural model estimation specifies the hypothesis tests as shown in Fig 3 and Table 6. The R^2^ values (squared multiple correlations) for US, AU, TTF and PI were found to be 0.055, 0.197, 0.363 and 0.314, respectively. Test results supported the six alternative hypotheses developed for this study. Hence it is evident that institutional factors positively estimate user satisfaction. So, the results supported H_1_ with β = 0.238, p <0.05. Similarly, user satisfaction positively predicts task technology fit (TTF), and the results of the tests advocate this hypothesis; hence, H_2_ is supported with β = 0.713, p <0.05, as reported in Table 6. It was analyzed that task technology fit had a significant effect on performance impact; therefore, H_3_ was also supported by the results with β = 0.262, p <0.05. Moreover, user satisfaction positively predicts actual usage of the system, and test results support this hypothesis β = 0.373, p<0.05, which is displayed in Table 6; hence H_4_ is also supported. As stated by H_5,_ actual usage of the system positively predicts performance impact, and the results of the tests support it, as shown in Table 6; so results support H_5_ with β = 0.200 and p<0.05.

**Table 6:** Evaluation of structural model.

Alternative Hypotheses	Relations	Estimate	S.E	C.R	p-value	Results
(Regression Coefficient)						
H_1_	US <—IF	0.238	0.043	5.295	***	Accept
H_2_	TTF <—US	0.713	0.066	10.719	***	Accept
H_3_	PI <—TTF	0.262	0.048	5.424	***	Accept
H_4_	AU <—US	0.373	0.063	5.914	***	Accept
H_5_	PI <—AU	0.200	0.060	3.348	***	Accept

*** p-value < 0.001

Variance accounted for (VAF) value was used to determine the efficacy of mediating effects. Complete mediation is regarded as the value of VAF higher than 80%, a value ranging from 20% to 80% is considered partial mediation, and a value less than 20% shows no mediation [131]. Table 7 study findings show partial mediation effects in the model. According to H_2a,_ institutional factors positively estimate task technology fit through mediating role of user satisfaction have indirect effect β = 0.170 and direct effect as β = 0.278, indicating partial mediation respectively. For the H_3a_ mediation test, tests similar to those stated above were performed indicating partial mediation effect, which represented that institutional factors firmly predict performance impact through the partial mediating role of user satisfaction and task technology fit in the sequence (β = 0.044) and β = 0.163 as indirect and direct effects respectively. For testing H_4a_, mediation tests similar to those mentioned before were performed, β = 0.089 was the indirect effect and β = 0.251 was the direct effect showing partial mediation. So, H_4a_ is supported, and hence institutional factors positively predict actual usage of the system through the partial mediating role of user satisfaction. For verification of H_5a,_ similar tests of mediation aforementioned were performed, and found indirect and direct effects β = 0.018 and β = 0.015, respectively. Therefore, H_5a_ is verified, so institutional factors positively predict performance impact through the partial mediating role of user satisfaction and actual usage of the system.

**Table 7:** Results of mediating effects.

Path	Direct Path	Indirect Path	Total Effect	VAF	Mediation Type
H_2a_ IF → US → TTF	0.278	0.170	0.448	37.94%	Partial
H_3a_ IF → US → TTF → PI	0.163	0.044	0.207	21.25%	Partial
H_4a_ IF→ US → AU	0.251	0.089	0.341	26.17%	Partial
H_5a_ IF→ US → AU → PI	0.015	0.018	0.033	54.54%	Partial

Hayes Process Macro for Moderation was utilized to test for moderation to test H_6_, which claims that cognitive absorption moderates the relationship between institutional factors and user satisfaction [132]. The test results are displayed in Table 8, where Coeff is the coefficient, SE represents the standard error, T represents the T-test, and the value of T should be greater than 1.96 to accept the alternative hypothesis. P is the significance value, and its value should be less than .05 to accept the alternative hypothesis. The last two columns, LLCI and ULCI, are the lower and upper intervals for the coefficient values, and they should not contain zero values to accept the alternative hypothesis. At first, the total direct effect of institutional factors was tested on user satisfaction; the output represented a significant interaction impact of institutional factors on user satisfaction (β = 0.749; t = 10.719; p< 0.001). Likewise, the direct effect of cognitive absorption as a moderator on user satisfaction was tested; the output represented a significant interaction impact of cognitive absorption on user satisfaction (β = 0.5014; t = 10.6868; p<0.001). Finally, we tested the interaction effect of institutional factors and cognitive absorption on user satisfaction; the output represented that cognitive absorption has a significant interaction impact on user satisfaction (β = -0.1566; t = 4.5098; p<0.001). Since the interaction term is crucial to determine the effect, the moderation effect exists in our framework, as shown in Table 8. Hence, the moderating role of cognitive absorption was found to be significant between institutional factors and user satisfaction, as shown in Table 8, supporting H_6_.

**Table 8:** Summarized results of moderating variable.

Variables	Coeff	SE	T	P-value	LLCI*	ULCI**
constant	4.6990	.0607	77.4241	.0000	4.5797	4.8183
IF—>US	.0749	.0457	1.6380	.1022	-.0150	.1649
CA—>US	.5014	.0469	10.6868	.0000	.4091	.5936
Interaction						
IF x CA—>US	.1566	.0347	4.5098	.0000	.0883	.2248

*Lower Limit Confidence Interval

** Upper Limit Confidence Interval

## 5. Discussion

Because student retention is a more difficult challenge for online courses than for face-to-face learning [28], therefore from the past few years, it has become a matter of great concern for higher education institutions and now, because of the outbreak of Covid-19 pandemic, this issue has become the prime concern for all the institutions in all the countries worldwide. As a result, it will be critical to investigate the factors contributing to student satisfaction and academic performance in online learning. Therefore this research investigated the link between institutional factors in terms of university support, technical assistance, instructor support and administrative on student satisfaction and performance in online learning during Covid-19.

In this research, an effort has been made to develop a model based on the consolidation of the Technology Acceptance Model (TAM), Task Technology Fit model (TTF), DeLone and McLean Model of Information Systems Success (DMISM) and Unified Theory of Acceptance and Use of Technology (UTAUT) model to examine the coalition between institutional factors, user satisfaction, TTF, actual usage, cognitive absorption, and performance impact by collecting and analyzing data from top public and private universities of Punjab, Pakistan.

According to the findings of this study, institutional factors have a significant positive impact on student satisfaction and performance. Participants of this study gave higher consideration to institutional factors, which validates the outcomes of the study by [133]. This study analyzed that institutional factors positively estimate user satisfaction. It deduced that the better the support of institutional factors in terms of administrative, technical, instructional, and technology support for the usage of the online learning system, the more probably the student attaining online education would consider that the service relates more to their demands, conceptions, behavior and way of life. Therefore, they will feel more complacent about choosing to depend on and attain online education. This finding supports previous research by [75, 79, 81]. Furthermore, [134] proposed that universities must provision students with adequate facilities and resources, and that technical assistance must be provided and shall be highly efficient in encouraging good perceptions and opinions among students. Apart from this, it was also exhibited that user satisfaction positively estimates task technology fit, which signifies that user satisfaction is a significant element in analyzing the success or failure of the new technology. It is also supported by the previous research findings [20]. This study further indicates that institutional factors positively estimate task technology fit with the mediating role of user satisfaction. This depicts that the more the students are content with the institutional factors support for online education technology, the more they will be satisfied with the services given by this technology, and the more the students will consider this technology fit to accomplish their requirements. Therefore more this technology would assist them in achieving their tasks [135, 136].

This study also infers that institutional factors significantly estimate performance impact by the mediating role of user satisfaction and task technology fit in series. Moreover, depending on the output of the empirical test carried out on the interrelationship between task technology fit and performance impact, it was observed that task technology fit positively estimates performance impact, an estimation that is similar to the conclusions of previous studies [84–88, 137]. This indicates that the more support provided by the institutional factors of the online learning system, the higher will be the level of satisfaction among the students with the facilities provided by the online education system in satisfying their needs, the more the students will observe the technology fit to accomplish their tasks, which will intensify academic performance and coursework productivity positively. However, the effectiveness and efficiency of student performance are enhanced due to task technology fit, and this technology fit fulfills the needs of the student when the student is more content, which is due to high support of institutional factors.

In addition to this, an interrelationship between user satisfaction and actual usage was analyzed, and it was observed that user satisfaction positively estimates the actual usage of the system. This output is also supported by the finding of previous research [98, 138]. This study also observed that institutional factors indirectly impact the actual usage of technology by the student via user satisfaction. So, the greater the support of institutional factors of the online learning system, the more the students will be content with the system and more the time span of the online learning usage by the students will increase [40, 139, 140].

This research also favors the hypothesis that actual usage of the system positively estimates the students’ performance impact. Few studies have enlightened the relationship between actual usage of system and performance impact, for example, in empirical research by [95], it was observed that actual system usage has a significant effect on the performance of an individual as they are using the system for accomplishing their task it will lead to the improvement in their performance. However, numerous studies refer to IS that emphasize that actual system usage has a significant positive effect on the performance of the system users [46, 78, 95, 98, 141]. This research also hypothesized that institutional factors estimate performance impact via the mediating role of user satisfaction and actual usage of the system. It infers that the higher the support of institutional factors of the online learning system is, the greater the level of satisfaction among the students, and as a result, the online learning system usage by the students will enhance. Hence, they will extend their time span of online learning system usage, which will have a positive impact on their academic excellence and coursework productiveness [19, 142–146]. So, this practice accommodates how the students are learning, which is regarded as an essential element in attaining their academic tasks.

It is further observed in this study that cognitive absorption plays the role of a moderator in the relationship that exists between institutional factors and user satisfaction depicts that if the students ascertain that the online learning system is satisfying their requirements and is valuable for them to carry out their academic tasks, then the students will be immensely content with it. Therefore, the more the level of cognitive absorption of the students, the greater their level of satisfaction will be. Numerous studies advocate the conception that cognitive absorption has a positive effect on user satisfaction [19, 110]

## 6. Theoretical and practical implications

This study can make various theoretical contributions. Firstly, this study facilitates the literature by analyzing the impact of cognitive absorption as a moderator on the interrelationship between institutional factors and user satisfaction. Secondly, this research further adds to the literature by exploring the process of sequence mediation from institutional factors to performance impact, where it is mediated by user satisfaction and task technology fit in series, and examining the effect of institutional factors on performance impact, where it is mediated by user satisfaction actual usage. However, this study is significant from a practical point of view because of various reasons. First, the e-learning method can significantly assist learning by efficient usage of time, and studying at a place of one’s convenience motivates acquiring education by utilizing minimal resources and minimizing spatial issues. As educational institutions in these covid-19 pandemic days rely more on providing online education as a precautionary measure, this research will be of much significance for the institutions and the students.

Secondly, this research aims to provide policymakers with an extensive framework that highlights the fact how the utilization of online learning technologies can elevate the academic performance of the students as educational institutions and governments throughout the world are making an effort to use online education at a huge level to assure that the students are provided with quality learning and education in this pandemic situation. According to the findings of the recommended framework, the academic performance of the students in online learning can be elevated if the institutional factors of the online system, constructs of user satisfaction, task technology fit, actual usage of the system and cognitive absorption are administered and regulated appropriately. Third, the anticipated outcome of this research will enable the students to improve and strengthen knowledge attainment, enhance educational performance, and improve students’ productive and innovative capabilities. It will minimize their stress level in attaining online education in the current situation of the covid-19 pandemic.

Even though Pakistan is a developing nation, it can still completely utilize the benefits offered by online education so that even with the scarce availability of resources, it can still impart high-quality education all over the country. Numerous governments have provided their students with modern technological gadgets; they have also reduced the cost of internet connections to significantly elevate the acquisition of online education in their respective countries. Pakistan can also productively utilize this strategy and support the nationwide use of online learning. Moreover, the proposed framework would significantly facilitate the instructors, the students, and other administrative staff of the universities to utilize the novel technologies for delivering online education in sorting out their numerous issues.

## 7. Conclusion

The World Health Organization (WHO) announced a pandemic of the novel SARS-CoV2 viral infection earlier this year, and since then, it has become the world’s most serious public health threat. To combat the pandemic, all countries around the world adopted a policy of social distancing, which resulted in the closing of educational institutions in most countries. All educational establishments were bound to make mandatory and suitable changes in their present educational framework and continue to deliver quality education to the students. Therefore, all the teaching and learning processes and sessions changed to e-learning. A comparative procedure was also carried out in Pakistan to avoid the spread of the pandemic. This unexpected shift from face-to-face classroom learning to online learning had put a lot of burden on the students, and that significantly influenced the students’ academic performance. The current study portrayed students’ viewpoints on online learning and identified factors that can help students improve their academic performance by implementing the most appropriate technology. This research recommended an integrated model between the (TAM) Technology Acceptance Model, (TTF) Task Technology Fit model, (DMISM) DeLone and McLean Model of Information Systems Success and (UTAUT) Unified theory of acceptance and use of technology model to resolve this problem. Institutional factors, task technology fit, user satisfaction, cognitive absorption, actual system use, and performance effect were all essential constructs in the framework.

The output of various tests indicated that the recommended framework effectively demonstrated the effects of online learning on students’ academic success. Furthermore, user satisfaction is highly significant for determining task technology fit and actual use of online learning. Furthermore, it facilitates the link between institutional factors and user satisfaction and actual usage and also the link between user satisfaction, actual usage, and performance impact. Besides, task technology fit is also important in evaluating academic performance and facilitating the relationship between user satisfaction and academic performance. Cognitive absorption is also essential in assessing user satisfaction. The output of the empirical tests performed significantly provided support to the associations between the framework constructs. The policymakers and educational experts should stress these features to elevate the likelihood of enhanced academic performance. Lastly, the findings of this study will effectively support the government of Pakistan’s plan and strategies related to the implementation of online education to create conditions that are compatible with student online learning assignments, social values, and lifestyles. In such a setting, students are more likely to use online learning to enhance their academic performance and, ultimately, the quality of their work-life.

## 8. Limitations and future research directions

Certain limitations have been observed in the current research that can anticipate a roadmap for future research. As data for this research was gathered from students of those universities situated in Punjab, it is being suggested that researchers who plan to conduct a similar study should also collect data from universities located in all provinces of Pakistan so that the outcomes of the current research can be generalized. Moreover, future research work can be carried out in a broader spectrum by analyzing the online education system implemented in various universities of Pakistan with the ones being implemented in universities of other developed countries. As the current research is constrained to the cross-sectional collection of data, researchers in the future should also observe longitudinal data. The researchers should also perform various experiments to compare the outcomes to make the current research framework more extensive. Apart from cognitive absorption, which is being utilized as a moderator in the present study, other moderators should also be considered by the researchers for future studies. Considering this viewpoint, perceived usefulness and perceived ease of use can be employed as a moderator, and further description relevant to this moderation effect can be further studied [45]. Moreover, numerous studies have analyzed that the human factor plays a significant role in influencing the students to acquire online education–like a research study by [147] proposes that transformational leadership can also be taken into consideration as a moderator to analyze relationships in online learning frameworks.

Furthermore, the relationship between the framework variables in the present can be presumed to formulate various scenarios. For instance, actual usage and user satisfaction can be replaced in future research work. Moreover, the present study is constrained only to the education sector, so for future studies, researchers can consider other disciplines as well, for instance, to carry out the assessment of the framework of the present research.

## Supporting information

S1 Appendix Questionnaire. (DOCX)

S2 Appendix Scale validity and reliability. (DOCX)

S1 File (SAV)
