# Supplementary material for: The role of institutional factors and cognitive absorption on students’ satisfaction and performance in online learning during COVID 19
Source: PLoS One. 2022 Jun 22;17(6):e0269609. doi: 10.1371/journal.pone.0269609 (PMC9216528; doi:10.1371/journal.pone.0269609)
Supplement: S1 Appendix — (DOCX) [file pone.0269609.s001.docx]

**Appendix A**

***QUESTIONNAIRE***

*Dear Respondent!*

*This study is being conducted by the Institute of Quality & Technology Management, University of the Punjab, Lahore. It aims to investigate “****The Role of Institutional Factors and Cognitive Absorption on Students’ Satisfaction and Performance in Online Learning during COVID 19****”. Your participation will be much appreciated and all data will be kept strictly confidential. During the questionnaire, if you feel offended in any manner, I sincerely apologize for the inconvenience of the matter caused.*

| **Gender** | Male | | | | Female | | | |
| --- | --- | --- | --- | --- | --- | --- | --- | --- |
| **Age** | 20 or less | | | 21-30 | | | 31-40 | |
| **University Name** |  | | | | | | | |
| **Education Level** | Intermediate | Bachelors | Masters | | M.Phil. | Ph.D. | | Others |
| **Email id** |  | | | | | | | |

**Record your responses on a scale given below:**

| **Strongly Disagree** | **Disagree** | **Somewhat Disagree** | **Neither Agree nor Disagree** | **Somewhat Agree** | **Agree** | **Strongly Agree** |
| --- | --- | --- | --- | --- | --- | --- |
| **1** | **2** | **3** | **4** | **5** | **6** | **7** |

| **INSTITUTIONAL FACTORS** | | | | | | | | | | | | | | | | | | |
| --- | --- | --- | --- | --- | --- | --- | --- | --- | --- | --- | --- | --- | --- | --- | --- | --- | --- | --- |
| 1 | | | | Interface with the institution (enrolling, assessment processes, evaluation, informing) is excellent | | | | | | 1 | 2 | | 3 | 4 | 5 | 6 | 7 |  |
| 2 | | | | Ability to construct timetables or schedules for learning activity/courses is acceptable. | | | | | | 1 | 2 | | 3 | 4 | 5 | 6 | 7 |  |
| 3 | | | | Help is available from university when I have a problem | | | | | | 1 | 2 | | 3 | 4 | 5 | 6 | 7 |  |
| 4 | | | | University tries to make my online classes as interesting as possible | | | | | | 1 | 2 | | 3 | 4 | 5 | 6 | 7 |  |
| 5 | | | | Overall, the information technology infrastructure is efficient | | | | | | 1 | 2 | | 3 | 4 | 5 | 6 | 7 |  |
| 6 | | | | Information was well structured/present | | | | | | 1 | 2 | | 3 | 4 | 5 | 6 | 7 |  |
| 7 | | | | Overall, the website was easy to use | | | | | | 1 | 2 | | 3 | 4 | 5 | 6 | 7 |  |
| 8 | | | | I knew where to ask for help when I had any technical issues. | | | | | | 1 | 2 | | 3 | 4 | 5 | 6 | 7 |  |
| 9 | | | | Technical support responded to my issues in a timely manner. | | | | | | 1 | 2 | | 3 | 4 | 5 | 6 | 7 |  |
| 10 | | | | I felt that I could ask any questions regarding the course materials to the instructor. | | | | | | 1 | 2 | | 3 | 4 | 5 | 6 | 7 |  |
| 11 | | | | The instructor provided clear instructions for assignments and quizzes. | | | | | | 1 | 2 | | 3 | 4 | 5 | 6 | 7 |  |
| 12 | | | | The instructor responded to students ‘questions in a timely manner. | | | | | | 1 | 2 | | 3 | 4 | 5 | 6 | 7 |  |
| 13 | | | | The feedback on my assignments was helpful. | | | | | | 1 | 2 | | 3 | 4 | 5 | 6 | 7 |  |
|  | | | | **USER SATISFACTION** | | | | | | | | | | | | | | |
| 13 | | | | My decision to use online learning was a wise one. | | | | | | 1 | 2 | | 3 | 4 | 5 | 6 | 7 |  |
| 14 | | | | The online learning has met my expectations. | | | | | | 1 | 2 | | 3 | 4 | 5 | 6 | 7 |  |
| 15 | | | | Overall, I am satisfied with online learning. | | | | | | 1 | 2 | | 3 | 4 | 5 | 6 | 7 |  |
| **TASK TECHNOLOGY FIT** | | | | | | | | | | | | | | | | | | |
| 16 | | | Online learning fits with the way I like to learn and study. | | | | | | | 1 | 2 | | 3 | 4 | 5 | 6 | 7 |  |
| 17 | | | Online learning is suitable for helping me complete my academic assignments. | | | | | | | 1 | 2 | | 3 | 4 | 5 | 6 | 7 |  |
| 18 | | | Online learning is necessary for my academic tasks. | | | | | | | 1 | 2 | | 3 | 4 | 5 | 6 | 7 |  |
|  | | | **PERFORMANCE IMPACT** | | | | | | | | | | | | | | | |
| 19 | | | Online learning helps me to accomplish my tasks more quickly | | | | | | | 1 | 2 | | 3 | 4 | 5 | 6 | 7 |  |
| 20 | | | Online learning makes it easier to complete my tasks. | | | | | | | 1 | 2 | | 3 | 4 | 5 | 6 | 7 |  |
| 21 | | | Online learning saves me money. | | | | | | | 1 | 2 | | 3 | 4 | 5 | 6 | 7 |  |
| 22 | | | Online learning improves my learning performance. | | | | | | | 1 | 2 | | 3 | 4 | 5 | 6 | 7 |  |
| 23 | Online learning enhances my academic effectiveness. | | | | | | | | | 1 | 2 | | 3 | 4 | 5 | 6 | 7 |  |
| 24 | Online learning helps reviews and eliminate errors in my work tasks. | | | | | | | | | 1 | 2 | | 3 | 4 | 5 | 6 | 7 |  |
| 25 | Online learning helps me to realize my future target. | | | | | | | | | 1 | 2 | | 3 | 4 | 5 | 6 | 7 |  |
| 26 | Online learning helps me acquire new knowledge. | | | | | | | | | 1 | 2 | | 3 | 4 | 5 | 6 | 7 |  |
| 27 | Online learning helps me acquire new skills. | | | | | | | | | 1 | 2 | | 3 | 4 | 5 | 6 | 7 |  |
| 28 | Online learning helps me to come up with innovative ideas. | | | | | | | | | 1 | 2 | | 3 | 4 | 5 | 6 | 7 |  |
|  | **COGNITIVE ABSORPTION** | | | | | | | | | | | | | | | | | |
| 29 | I often spend more time on the Web than I had intended | | | | | | | | | 1 | 2 | | 3 | 4 | 5 | 6 | 7 |  |
| 30 | While on the Web, I am immersed in the task I am performing. | | | | | | | | | 1 | 2 | | 3 | 4 | 5 | 6 | 7 |  |
| 31 | I enjoy using the Web. | | | | | | | | | 1 | 2 | | 3 | 4 | 5 | 6 | 7 |  |
| 32 | The Web allows me to control my computer interaction. | | | | | | | | | 1 | 2 | | 3 | 4 | 5 | 6 | 7 |  |
| 33 | Interacting with the Web makes me curious. | | | | | | | | | 1 | 2 | | 3 | 4 | 5 | 6 | 7 |  |
| 34 | I like to experiment with new information technologies. | | | | | | | | | 1 | 2 | | 3 | 4 | 5 | 6 | 7 |  |
|  | | | | **ACTUAL USAGE** | | | | | | | | | | | | | | |
| 35 | | | | On average, how frequently do you use online learning? | | | | | | | | | | | | | | |
| 1. Certainly not | | | | | 2. Less than once a month | 3. Once a month | 4. A few times a month | 5. A few times a week | 6. About once a day | | | 7. Several Times a day | | | | | | |
| 36 | | Average, how much time do you spend per week using online learning? | | | | | | | | | | | | | | | | |
| 1.Certainly not | | | | | 2. Almost never | 3. Less than two hours | 4. Two to four hours | 5. Four to six hours | 6.Six to eight hours | | | 7.More than eight hours | | | | | | |
