# Supplementary material for: The role of institutional factors and cognitive absorption on students’ satisfaction and performance in online learning during COVID 19
Source: PLoS One. 2022 Jun 22;17(6):e0269609. doi: 10.1371/journal.pone.0269609 (PMC9216528; doi:10.1371/journal.pone.0269609)
Supplement: S2 Appendix — (DOCX) [file pone.0269609.s002.docx]

**Appendix B**

**Scale validity and reliability**

|  |  | |  | | **Standardized Estimate** | **Cronbach Alpha** |
| --- | --- | --- | --- | --- | --- | --- |
|  |  | | PI | |  | .979 |
| PI1 | <--- | | PI | | 0.922 |  |
| PI2 | <--- | | PI | | 0.89 |  |
| PI3 | <--- | | PI | | 0.923 |  |
| PI4 | <--- | | PI | | 0.937 |  |
| PI6 | <--- | | PI | | 0.937 |  |
| PI7 | <--- | | PI | | 0.935 |  |
| PI8 | <--- | | PI | | 0.913 |  |
| PI9 | <--- | | PI | | 0.896 |  |
| PI10 | <--- | | PI | | 0.889 |  |
|  |  | | IF | |  | .952 |
| IF1 | <--- | | IF | | 0.726 |  |
| IF2 | <--- | | IF | | 0.878 |  |
| IF4 | <--- | | IF | | 0.848 |  |
| IF5 | <--- | | IF | | 0.821 |  |
| IF6 | <--- | | IF | | 0.813 |  |
| IF7 | <--- | | IF | | 0.8 |  |
| IF8 | <--- | | IF | | 0.787 |  |
| IF9 | <--- | | IF | | 0.776 |  |
| IF10 | <--- | | IF | | 0.748 |  |
| IF12 | <--- | | IF | | 0.795 |  |
| IF13 | | <--- | | IF | 0.792 |  |
|  | | CA | |  |  |  |
| CA1 | | <--- | | CA | 0.847 |  |
| CA2 | | <--- | | CA | 0.955 |  |
| CA4 | | <--- | | CA | 0.866 |  |
| CA6 | | <--- | | CA | 0.975 |  |
|  | |  | | TTF |  |  |
| TTF1 | | <--- | | TTF | 0.957 |  |
| TTF2 | | <--- | | TTF | 0.895 |  |
| TTF3 | | <--- | | TTF | 0.937 |  |
|  | |  | | US |  |  |
| US1 | | <--- | | US | 0.82 |  |
| US2 | | <--- | | US | 0.878 |  |
| US3 | | <--- | | US | 0.934 |  |
|  | | AU | |  |  |  |
| AU1 | | <--- | | AU | 0.871 |  |
| AU2 | | <--- | | AU | 0.716 |  |
